# The Avon Longitudinal Study of Parents and Children - A resource for COVID-19 research: Generation 2 questionnaire data capture May-July 2020

**DOI:** 10.12688/wellcomeopenres.16414.2

**Published:** 2021-04-14

**Authors:** Daniel Smith, Kate Northstone, Claire Bowring, Nicholas Wells, Michael Crawford, Rebecca M. Pearson, Amy Thomas, Ellen Brooks-Pollock, Deborah A. Lawlor, Nicholas John Timpson

**Affiliations:** 1 ALSPAC, Department of Population Health Sciences, Bristol Medical School, University of Bristol, Bristol, BS8 2BN, UK; 2 MRC Integrative Epidemiology Unit, University of Bristol, Bristol, UK; 3 Population Health Sciences, Bristol Medical School, University of Bristol, Bristol, BS8 2BN, UK; 4 NIHR Bristol Biomedical Research Centre, Bristol, UK; 5 Bristol Veterinary School, University of Bristol, Bristol, BS40 5DU, UK

**Keywords:** ALSPAC, Children of the 90s, Cross-generation, COVID-19, Coronavirus, Children, Mental Health, Contact Patterns

## Abstract

The Avon Longitudinal Study of Parents and Children (ALSPAC) is a prospective population-based cohort study which recruited pregnant women in 1990-1992 from the Bristol area (UK). ALSPAC has followed these women, their partners (Generation 0; G0) and their offspring (Generation 1; G1) ever since. From 2012, ALSPAC has identified G1 participants who were pregnant (or their partner was) or had become parents, and enrolled them, their partners, and children in the ALSPAC-Generation 2 (ALSPAC-G2) study, providing a unique multi-generational cohort. At present, approximately 1,100 G2 children (excluding those *in utero*) from 810 G1 participants have been enrolled.

In response to the COVID-19 pandemic, ALSPAC rapidly deployed two online questionnaires; one during the initial lockdown phase in 2020 (9 ^th^ April-15 ^th^ May), and another when national lockdown restrictions were eased (26 ^th^ May-5 ^th^ July). As part of this second questionnaire, G1 parents completed a questionnaire about each of their G2 children. This covered: parental reports of children’s feelings and behaviour since lockdown, school attendance, contact patterns, and health. A total of 289 G1 participants completed this questionnaire on behalf of 411 G2 children.

This COVID-19 G2 questionnaire data can be combined with pre-pandemic ALSPAC-G2 data, plus ALSPAC-G1 and -G0 data, to understand how children’s health and behaviour has been affected by the pandemic and its management. Data from this questionnaire will be complemented with linkage to health records and results of biological testing as they become available. Prospective studies are necessary to understand the impact of this pandemic on children’s health and development, yet few relevant studies exist; this resource will aid these efforts.

Data has been released as: 1) a freely-available dataset containing participant responses with key sociodemographic variables; and 2) an ALSPAC-held dataset which can be combined with existing ALSPAC data, enabling bespoke research across all areas supported by the study.

## Introduction

As of 30 ^th^ March 2021, the coronavirus disease 2019 (COVID-19) pandemic continues to be a rapidly developing global health challenge. Understanding the prevalence of this disease, as well as the social, demographic and environmental factors shaping infection, disease progression and mental and physical health response, requires detailed studies, ideally with prospective data. Of particular importance is the effect of the pandemic on children, especially in relation to their physical and mental development, mental health and well-being ^1– 6^. The impact of COVID-19 infection on children’s acute physical health may be minimal; while children are able to become infected with COVID-19, the majority are either asymptomatic or show mild symptoms, with only a minority of cases progressing to a severe form of the disease ^7– 9^. However, long-term effects in children and adults are emerging and are currently under-researched. Furthermore, children’s physical and mental health may be impacted through the management of the pandemic (e.g., school closures, physical distancing and limited outdoor activities) and by the conditions found around them in the home environment, for example by reducing opportunities for physical activity, not seeking health care for non-COVID illness or accidents, and missing planned immunisations ^2, 3, 6, 10– 13^.

Consequently, there have been calls for research to explore the impact of the pandemic on children’s health and development as a matter of urgency to help inform and develop public health responses and mitigation strategies ^4, 11^. For instance, previous work during emergencies and disasters has demonstrated that a lack of routine and social isolation can have a detrimental impact on children’s mental health and well-being ^14, 15^. Despite this, current mental health research on children in response to the COVID-19 pandemic is limited, and the majority of existing studies in this area ^5^ have utilised cross-sectional work which may be subject to bias when comparing changes relative to pre-pandemic behaviours and mental health (e.g., recall bias; although see 16 for a longitudinal study). As such, there is a need for prospective studies – both in terms of mental health and wider health and development – to examine responses to the COVID-19 pandemic more robustly and corroborate conclusions from cross-sectional studies.

The Avon Longitudinal Study of Parents and Children (ALSPAC) is a unique three-generational study, comprising ‘G0’: the cohort of original pregnant women, the biological father and other carers/partners; ‘G1’: the cohort of index children; and ‘G2’: the cohort of offspring of the index children. The study has a wealth of existing biological, genetic and phenotypic data across these generations ^17– 20^. Using our infrastructure for online data collection, ALSPAC has been well-placed to capture information across key parts of the population in light of the COVID-19 pandemic, from those in middle/old age (the G0 cohort; mean age ~59 years), those in early adulthood (the G1 cohort; mean age ~28 years), and children (the G2 cohort; mean age ~3.5 years). ALSPAC is therefore uniquely-placed to contribute to the understanding of COVID-19 and its management on the behaviour and development of children, as the study has data not only for children (generation G2), but also for the entire lives of at least one of their parents (generation G1) and for the past ~30 years of their grandparents (generation G0).

The wider COVID-19 data collection in ALSPAC will include data from three main sources: self/parental-reported data from questionnaires, data from clinical services based on linkage to health records, and information from biological samples collected during the pandemic. The data from these sources are intended to be complementary and help address different potential research questions around COVID-19 and its management.

This data note describes the data collected via our second online questionnaire which focussed on G2 children and was completed between 26 ^th^ May and 5 ^th^ July 2020. It provides a summary of the responses given by G1 participants about their G2 children’s health and well-being. To describe potential sources of selection bias we also present results showing the associations of some key sociodemographic characteristics with G2 questionnaire completion.

## Methods

### Setting

ALSPAC is a multi-generation longitudinal cohort that recruited pregnant women residing in the former county of Avon, UK with expected dates of delivery 1 ^st^ April 1991 to 31 ^st^ December 1992 ^17, 18^. The initial cohort consisted of 14,541 pregnancies resulting in 14,062 live births and 13,988 children who were alive at 1 year of age. From the age of seven onwards, the initial sample was bolstered with eligible cases who had originally failed to join the study (i.e., children born in the Avon area during the birth years of the ALSPAC-G1 participants who not were recruited during their mother’s pregnancy); following this further recruitment there were subsequently 14,901 children alive at 1 year of age ^19^. Please note, the study website contains details of all the data that is available through a fully searchable data dictionary and variable search tool ( http://www.bristol.ac.uk/alspac/researchers/our-data).

Recruitment of G1 participants and their G2 offspring into the ALSPAC-G2 study began on 6 ^th^ June 2012. Rather than being a birth cohort with a set date of birth and location criteria (as with recruitment of G1 offspring during pregnancy), ALSPAC-G2 is an open cohort which recruits G2 children at any age ^20^. To date, approximately 50% of G2 children have been recruited during their mother’s pregnancy, with over 80% of children recruited before the age of three; the number recruited during the mother’s pregnancy continues to increase over time. Repeated socioeconomic, psychological, developmental, health and anthropometric data and biological samples are collected from G2 children, the G1 parent, and the partner of the G1 parent via questionnaires, face-to-face clinics and information from health records (see 20 for further details). As of 18 ^th^ June 2020, 1,116 G2 children (527 female [47%]; 589 male [53%]; excluding those *in utero*), 810 G1 participants (603 female [74%]; 207 male [26%]), and 418 G1 partners (126 female [30%]; 292 male [70%]) are enrolled in the ALSPAC-G2 study. Of these 1,116 G2 children, 455 (41%) are from families with only one child enrolled in ALSPAC-G2, 476 (43%) have one other sibling enrolled, 153 (14%) have two other siblings enrolled, and 32 (3%) have three or more other siblings enrolled. In nearly every instance where siblings are known, all siblings have been enrolled into ALSPAC-G2 (with fewer than a handful of known exceptions). However, it is important to note that later-born siblings would remain unknown to ALSPAC if contact is lost with their parents after enrolment.

In response to COVID-19 it was necessary to develop a data collection strategy which was practical, would yield data quickly and could be updated and repeated if necessary. For these reasons, we chose to use an online only data collection approach, restricting our invites to those participants with a valid email address (and coordinated with a systematic communications/outreach campaign to obtain updated information from participants). The questionnaire was deployed using REDCap (Research Electronic Data CAPture tools); a secure web application for building and managing online data collection exercises, hosted at the University of Bristol ^21^. The development of the first and second G0/G1 COVID questionnaires are described elsewhere ^22, 23^.

### Content design

Content was developed primarily to answer questions about the impact of COVID-19 on children’s feelings and behaviours (including mental health and behavioural/emotional difficulties), contact patterns, and COVID-related health. Many of the questions related to children’s mental health and behaviour (discussed in more detail below) were chosen to be identical to pre-pandemic G2 data collections, thus permitting longitudinal analyses to assess the impact of the pandemic on children’s mental well-being, their behaviour and G1 parenting practices. In addition to questions about their G2 child’s mental health and behaviours, parents were also asked to report whether their child had experienced COVID-19, symptoms that might indicate COVID-19 infection, and recent contact patterns. The focus on mental health and behaviours in this questionnaire was to minimise G1 questionnaire burden and because information on other outcomes – such as changes in patterns of health-seeking behaviour, new diagnoses, and management of new or existing health problems – could be obtained from other sources, such as record linkage and biological samples.

Whilst ALSPAC is a unique multi-generational study, our collection of COVID-19 data has been done in collaboration with other population studies where appropriate. The questionnaire data (across generations) was co-developed by ALSPAC as part of the Wellcome Trust’s Longitudinal Population Study (LPS) COVID-19 Steering Group and Secretariat, a network of UK and international longitudinal population studies (see http://www.bristol.ac.uk/alspac/researchers/wellcome-covid-19/). This means, where appropriate, analyses can be done in collaboration with other cohorts in order to facilitate replication and increase statistical power. For example, combining data from ALSPAC-G0, ALSPAC-G1, and Generation Scotland, we have shown that the COVID-19 pandemic and/or its management appears to worsen mental health in adults and have identified groups at increased risk of COVID-19 related depression and anxiety ^24^. However, this G2 questionnaire data was developed in-house by ALSPAC (in collaboration with other studies where appropriate; see below for more details), meaning that many questions differ from those in the Wellcome LPS COVID-19 questionnaire, as the number of child-based questions available were small. These decisions were made based on the need to repeat measures previously collected as part of ALSPAC-G2 to facilitate longitudinal analyses, in addition to the need to collect data not part of the Wellcome questionnaire (e.g., in-depth child contact patterns), while at the same time minimising participant burden as much as possible. Nonetheless, we are open to collaboration and are keen to harmonise data collections with other cohorts to facilitate co-ordinated analyses.

The ALSPAC-G2 questionnaire was embedded within the second ALSPAC COVID questionnaire sent to all G0 and G1 participants, including enrolled G1 partners ^23^. G1 participants enrolled as parents in the ALSPAC-G2 study were asked to complete the ‘Your Children’ section of this questionnaire (section F), which detailed the number of children the participant has and their date of births. These participants were then asked to complete the G2 questionnaire for each of their children, with the questions tailored depending on the child’s age. For the questions about contact with other children and adults (section 3), parents were asked to complete the questionnaire with help from their children.

The G2 questionnaire included four sections, and captured information on the following:

1. Children’s feelings and behaviour (including mental health)
  ● For children aged 0–3 years, the Carey Infant Temperament Questionnaire ^25^ was used to assess the temperament/behavioural style of the child (this is also asked at age 6 months as part of the wider ALSPAC-G2 study and was also collected for G1 participants when they were the same age). Note that for this G2 COVID questionnaire only the ‘mood’ and ‘distractibility’ subscales were asked.
  ● For children aged 3 and above, the Revised Rutter Parent Scale for Pre-school Children ^26^ was used to assess child mental health and behavioural/emotional problems (this is also asked at age 48 months as part of the wider ALSPAC-G2 study and was also collected for G1 participants when they were the same age)
  ● Child and parental worries about COVID-19 (with different questions tailored to the child’s age, with parents of children aged 0–3 shown one set of questions and parents of children aged 3 or older shown a different set; these questions were adapted from the Co-SPACE study: https://www.psy.ox.ac.uk/research/topic-research-group/supporting-parents-adolescents-and-children-during-epidemics)
  ● Whether the child has a regular routine (these questions were adapted from the Co-SPACE study)
  ● Child temper tantrums and parent’s response to bad behaviour (this is also asked at ages 36 months and older as part of the wider ALSPAC-G2 study and was also collected for G1 participants when they were approximately the same age; non-standardised measures previously developed by ALSPAC)
  ● Parental conversations with child about COVID-19 and current events (these questions were adapted from the Co-SPACE study)

2. School
  ● Whether the child is currently attending school, pre-school or nursery (non-standardised measure)
  ● If the child is not going to school, what they like about not going to school and what they miss about not going to school (non-standardised measure)

3. Contacts (completed with help from their G2 child)
  ● Social contacts and methods of communication (these questions were adapted from the Co-SPACE study)
  ● Time spent with various family members and friends (also asked in all ALSPAC-G2 pre-pandemic questionnaires; non-standardised measure previously developed by ALSPAC)
  ● Number, context and age of social contacts that child had yesterday (both in groups and with individuals; based on the Social Contact Survey ^27^)

4. COVID-19 related health
  ● Whether the child had/has COVID-19 (non-standardised measure)
  ● Symptoms of COVID-19 and negative control symptoms since March 2020 (adapted from the Wellcome LPS COVID-19 questionnaire)
  ● Missed vaccinations as a result of COVID-19 (non-standardised measure)

The final questionnaire used is available with the associated data dictionary (which includes frequencies of all variables that are available) and both can be accessed in the *Extended data*.

### Invitation and reminder strategy

Between the 26 ^th^ and 29 ^th^ May 2020, all participants (G0, G1 and G1 partners enrolled as part of G2) for whom we had an active email address were sent an invitation to complete the second COVID questionnaire (see 23), with additional invites sent out on 10 ^th^, 19 ^th^ and 26 ^th^ June, as a result of outreach work undertaken by the ALSPAC team. Only original G1 participants enrolled as parents in the ALSPAC-G2 study were asked to complete the G2 part of the questionnaire; G1 partners and G0 participants were not asked to complete the G2 questionnaire and were not shown the ‘Your Children’ section of the G0/G1 questionnaire. If both parents were original G1 participants, as occurs for approximately 9% of ALSPAC-G2 pregnancies/children ^20^, then it was possible for duplicate data to be collected on behalf of a G2 child. However, for this G2 COVID questionnaire only two G1 parents (linked to three G2 children) gave data about the same child (see the ‘response rate’ section for more details).

Participants were not contacted if our administrative database records indicated that they or their G2 child were deceased, had withdrawn from the study, had declined further contact, had declined questionnaires or for safeguarding reasons. Of 810 G1 parents enrolled in ALSPAC-G2, 60 participants (7%) were not invited to complete this questionnaire; 39 due to not having a valid email address, and 21 for an administrative reason listed above. The questionnaire survey was live on the online platform for just over one month. On the 11 ^th^ and 12th June, non-responders were sent a reminder email to complete the questionnaire. Finally, reminders were sent on 26 ^th^ June to those participants who had previously completed our first COVID questionnaire but had not yet responded to the second.

In addition, traditional (print, radio, tv) and social media (Facebook, Instagram and Twitter) were used to inform participants that the questionnaire was live, asking them to contact us if they had not received it and to encourage completion. These communication channels were also used to encourage re-engagement of friends and family back into the study. Unlike our standard questionnaires (usually completed annually) we did not provide any incentive for completion; however, we did offer a prize draw (three prizes of £100) for those who completed their questionnaire by 29 ^th^ June.

### Response rate

**Figure 1.**
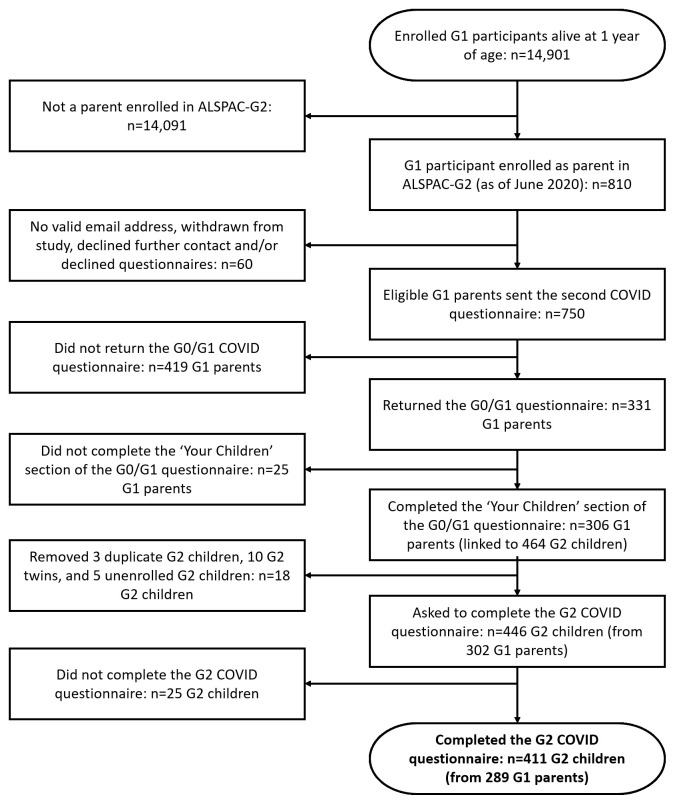
Flow diagram describing the study numbers of those with G2 COVID questionnaire data.

A total of 6,148 invitations were sent out to G1 participants, of which 750 (12%) were enrolled as parents in ALSPAC-G2 ( Figure 1). Of these 750 parents, 331 (44%) returned the main G0/G1 questionnaire, a response rate identical to that of G1s not enrolled in ALSPAC-G2 (44%; 2,380/5,398). While this response rate is lower than the G1 response rate to the first ALSPAC COVID-19 questionnaire (51%) ^22^, it is typical of other recent G1 questionnaires with response rates ranging between 42% and 48%. Of these 331 parents who returned a G0/G1 questionnaire, 306 (92%; 41% of eligible parents) completed the ‘Your Children’ section of the questionnaire and therefore were presented with the G2 questionnaire based on the number of children they said they had. As with the ALSPAC-G0/G1 COVID questionnaires ^22, 23^, female G1 parents were more likely to respond than male G1 parents ( Table 1).

**Table 1.** Number of eligible/invited G1 parents who responded to the ‘Your Children’ section of the questionnaire. Note that 25 G1 parents began the G0/G1 questionnaire but did not complete the ‘Your Children’ section.

Cohort Group	Eligible/ Invited ^1^	Responded to the ‘Your Children’ section (if eligible/invited)
Male G1 parents	174	47 (27%)
Female G1 parents	576	259 (45%)
**TOTAL**	**750**	**306 (41%)**

^1^ valid email address, marked as contactable for questionnaires, and enrolled in ALSPAC-G2 as a parent

Of these 306 parents who gave details about their children, 179 (59%) said that they only had one child, 98 (32%) said they had two children, 27 (9%) said they had three children, and two (1%) said they had four children, giving a total of 464 G2 children. Three of these 464 children were found to be duplicates with data provided by both parents, so one questionnaire response for each of these was removed from the dataset (the data from the G1 parent who completed the questionnaire first was kept, and the other dropped). A further 10 children were removed from the final dataset because they were twins; linking of G2 children who were the subject of this questionnaire to their existing data was done using their parent’s ALSPAC IDs and the G2 child’s date of birth. Unfortunately, this meant that it was not possible to link twins’ data back to their unique G2 child ID, and hence pre-pandemic ALSPAC-G2 data, with any certainty (note that this issue of linking twin data is only a problem for this COVID questionnaire and is because the questionnaire was embedded within a general G1 questionnaire; in all other ALSPAC-G2 data collections twin data is uniquely identified by a G2 child ID). Five further G2 children were removed as the child was previously unknown to ALSPAC and it was not possible to enrol them in the ALSPAC-G2 study (reasons included: foster child; step-child and biological parent not enrolled in ALSPAC-G2; unable to include child due to safe-guarding measures; unable to contact parent to enrol new G2 child; and child not born yet).

A flow chart of this process is displayed in Figure 1, resulting in a final dataset containing records of 446 unique G2 children from 302 G1 parents. Four-hundred and eleven of these G2 children (from 289 parents) have data from the G2 questionnaire (92%). When split by child number (the order in which parents entered their children’s dates of birth in the ‘Your Children’ section of the G0/G1 questionnaire), questionnaire fatigue was apparent, with the proportion of questionnaire responses higher for earlier children ( Table 2). Although G1 parents were free to enter their children’s date of births in any order, most entered them in date order (oldest first). Of parents with more than one G2 child in the final dataset, on average the second child was 3.1 years younger than the first child (SD = 1.9; range = -7.4 to 2.7; n = 118), the third child was an average of 5.9 years younger than the first child (SD = 2.8; range = -9.6 to 3.8; n = 24), while the fourth child was an average of 7.1 years younger than the first (SD = 0.3; range = -7.3 to -6.9; n = 2). As these later children are less likely to have completed questionnaire data ( Table 2), younger siblings are therefore less likely to have data than older siblings.

**Table 2.** ALSPAC-G2 COVID-19 questionnaire completion, split by child number. This table only includes information for those G2 children where the G1 parent completed the ‘Your Children’ section of the G0/G1 questionnaire, after removal of duplicate G2 children, G2 twins, and unenrolled G2 children (n = 446 G2 children; n = 302 G1 parents; see Figure 1).

Child number	Total number of children	Completed G2 questionnaire (%)	Not completed G2 questionnaire (%)
Child 1	300	287 (96%)	13 (4%)
Child 2	119	102 (86%)	17 (14%)
Child 3	25	22 (88%)	3 (12%)
Child 4	2	0 (0%)	2 (100%)
**TOTAL**	**446**	**411 (92%)**	**35 (8%)**

## Key results

### Sociodemographic characteristics

Characteristics of responders according to key sociodemographic variables that will be released with the complete dataset can be seen in Table 3. G2 child ages ranged from 0 to 13 years with a mean of 3.4 (SD = 3.1), with an approximately even split of children who were younger than three years (51%) and three years and older (49%). There was a slight bias towards male G2 children (52%). Consistent with responses to the G0/G1 COVID2 questionnaire, the sample of G1 parents is predominantly white (98%; however, note that the ethnicity of the G1 parent’s partner, if not a G1 participant, and hence the G2 child’s ethnicity, is unknown). Fewer G1 parents had at least A level qualifications, compared to the wider G1 sample (53% in the G1 parent sample vs 79% in the wider G1 sample; see Table 2 of 23). For an assessment of potential biases in parents who completed the questionnaire, compared to the wider G2 parent cohort, see the section ‘Assessment of potential selection bias’ below.

**Table 3.** Summary of key characteristics for those who responded to the G2 COVID questionnaire. n (%) for categorical variables or mean (sd) for continuous variables. Note that where G1 parents have more than one child in the dataset, G1 characteristics are only counted once. *n* = 411 for G2 child data; *n* = 289 for G1 parent data (although data for some G1 parent covariates are incomplete, hence why sample sizes may vary).

Key Characteristic ( *n*)	Value
G2 child age (years; *n* = 411)	3.4 (3.1)
G2 child age category ( *n* = 411) < 3 years old 3 or more years old	210 (51%) 201 (49%)
G2 child sex ( *n* = 411) Male Female	214 (52%) 197 (48%)
G1 parent age (years; *n* = 289)	27.9 (0.6)
Sex of G1 parent completing questionnaire ( *n* = 289) Male Female	43 (15%) 246 (85%)
G1 parent age at G2 child’s birth (years; *n* = 289)	24.3 (3.1)
Latest G1 parent BMI ( *n* = 276) ^1^	26.5 (6.1)
Latest G1 parent Systolic BP ( *n* = 268) ^1^	114.3 (10)
Latest Diastolic BP ( *n* = 268) ^1^	67.9 (7.6)
G1 parent education level ( *n* = 249) ^2^ GCSE/Lower Vocational AS/A level Degree/Higher	49 (20%) 67 (27%) 55 (22%) 78 (31%)
G1 parent ethnicity ( *n* = 243) ^3^ White Non-white	237 (98%) 6 (2%)

^1^Data taken from the most recent clinic that individual attended (where available) ^2^Data taken from most recent questionnaire for G1 (where available) ^3^Data taken from G0 mother pregnancy questionnaire (where available)

### COVID-19 symptoms and diagnoses

Parents were asked to report the COVID-19 status of their child, with the responses ‘Yes, diagnosed by a doctor and recovered’, ‘Yes, diagnosed and still ill’, ‘Suspected and recovered’, ‘Suspected and still ill’, and ‘No’. Of the 394 G2 children with data for this question, 41 (10%) were suspected of having had COVID-19 and had since recovered. All other 353 (90%) responses were ‘No’, meaning that no children had had COVID-19 diagnosed by a doctor or were still suspected to be ill with COVID-19 at the time of questionnaire completion. The lack of children with a doctor diagnosis is not surprising as from the start of management of the pandemic government advice was clear that no-one should go to a health care provider if they displayed symptoms, and instead should isolate at home. As a result, across all ages in the population a doctor diagnosis is only available if their symptoms were severe enough to be admitted to hospital, and hospitalisation for COVID-19 is extremely rare in children.

Children under three years of age were slightly more likely to be suspected of having had COVID-19, compared to children aged three or over (25/205 [12%] of those under 3 vs 16/189 [8%] of those 3 and older; Table 4). By comparison, of the 286 G1 parents who answered the equivalent question in the G0/G1 questionnaire (mean age 27.9 years), 11 (4%) either had a confirmed positive test or were suspected by a doctor of having COVID-19, 37 (13%) had their own suspicions that they had COVID-19, while 238 (83%) did not think that they had COVID-19. Self-reported COVID status in G1 parents was broadly similar to that among the 2,404 non-parent G1 participants (64 [3%] said ‘confirmed positive test or suspected by doctor’, 363 [15%] said ‘own suspicions’, and 1,977 [82%] said ‘no’), suggesting that parents were not at greater risk of contracting COVID-19.

**Table 4.** Associations between potential risk factors and parent-reported G2 COVID-19 status. n (%) for categorical variables or mean (sd and n) for continuous variables. For all analyses, standard errors were clustered on parent ID to account for non-independence of data due to clustering within families.

		G2 child had COVID-19 (parent- reported)		Unadjusted odds ratio (95% CI)
		No	Suspected	
**G1 parent had COVID-19** **(self-reported)**	No	317 (97%)	10 (3%)	30.7 (11.9; 79.1)
	Yes, positive test OR doctor suspected OR own suspicions	32 (51%)	31 (49%)	
**Parent education**	GCSE/Vocational	152 (92%)	14 (8%)	1.5 (0.65; 3.55)
	A level/Degree	150 (88%)	21 (12%)	
**Parent age (months)**		340.7 (SD = 5.8; n = 353)	339.3 (SD = 6.4; n = 41)	0.96 (0.89; 1.04)
**Parent ethnicity ^1^**	White	***	***	1.3 (0.19; 8.71)
	Non-white	***	***	
**Child sex**	Male	188 (90%)	20 (10%)	1.2 (0.67; 2.14)
	Female	165 (89%)	21 (11%)	
**Child age**	< 3 years old	180 (88%)	25 (12%)	0.67 (0.33; 1.34)
	3 years or older	173 (92%)	16 (8%)	
**Child attending school/** **pre-school/nursery**	No	196 (90%)	33 (10%)	1.16 (0.5; 2.72)
	Yes	54 (89%)	7 (11%)	
**Child met any groups or** **2 or more yesterday**	No	304 (91%)	31 (9%)	1.92 (0.83; 4.45)
	Yes	46 (84%)	9 (16%)	
**Number of individual contacts yesterday ^2^**		3.2 (SD = 2; n = 255)	3.7 (SD = 2.2; n = 31)	1.1 (0.94; 1.3)

^1^ Note that due to small and potentially disclosive cell counts for parents with a non-white ethnicity (cell count < 5), the raw numbers for ‘parent ethnicity’ have been withheld. ^2^ Note that the number of individual contacts excludes individuals met as part of a group. Additionally, in the whole G2 sample seven responses were of ‘11 or more’; here these have been treated as having 11 contacts.

Table 4 details the factors associated with parent-reported COVID-19 status in G2 children (‘suspected’ vs ‘no’). These results were obtained from unadjusted logistic regression models with standard errors clustered on parent ID (to account for the non-independence of data due to clustering within families). We found a strong association between the G1 parent’s self-reported COVID-19 status (with ‘report of positive test’, ‘doctor suspected’, and ‘own suspicions’ coded as a positive case) and the G2 child suspected of having COVID-19 (odds ratio = 30.7, 95% CI: [11.9; 79.1]). There is evidence that children of parents with higher educational attainment, children younger than 3, and children who had more social contacts on the previous day (either in groups of two or more or based on the total number of individual contacts) were more likely to have parent-reported symptoms (although in all of these analyses the 95% confidence intervals include the null). None of the other factors in Table 4 displayed a clear association with parent-reported G2 COVID-19 status. It is important to stress that these results illustrate the potential of the ALSPAC-G2 data, including the precision with which child associations for relatively rare outcomes can be estimated. We feel that this is helpful for a Data Note. We have not undertaken adjusted analyses to explore specific research questions that we anticipate future users of these data will address.

Parents also completed a 22-item monthly symptom checklist detailing their child’s health since official lockdown was announced in the UK (23 ^rd^ March 2020). For the adult (G0 and G1) COVID questionnaires ^23^ these symptom checklists were used to predict COVID-19 cases based on the algorithm derived by Menni and colleagues ^28^. However, children infected with COVID-19 are thought to display different symptomatology, including fewer of the symptoms that were initially associated with COVID-19 in adults, more gastrointestinal problems, a higher frequency of rashes, reduced coughing and less shortness of breath ^7, 29, 30^. As such, and together with the current lack of any standard diagnostic criteria in children, we have not attempted to predict COVID-19 cases from symptoms in this dataset; future work, especially in combination with serological testing and linkage to health records, will address this question in greater detail. These G2 child symptoms can also be combined with the G1 parental symptoms to explore family-level constellations of symptoms and COVID-19 infections.

### Mental health and behavioural characteristics

For children aged less than three years of age, child temperament was assessed using 19 items from the ‘mood’ and ‘distractibility’ sub-scales of the Carey Infant Temperament Questionnaire ^25^. Of 210 children aged younger than three, 197 (94%) had seven or fewer missing items on this scale, and 157 of these 197 (80%) had no missing data. The total Carey infant difficulties score for children with complete data was 29.8 (SD = 7.2; range = 11 - 44), of out a maximum possible 76. The prorated score for all 197 children with seven or fewer missing items was 30.2 (SD = 7.3; range = 11 – 47.5), and was calculated by taking the mean score of all items with data and then multiplying by the total number of items in the scale (19). Children with missing data had on average a slightly higher temperament score (mean = 31.7; SD = 7.6; n = 40) than children with complete data; a difference of 1.96 (95% CI: [-0.6; 4.5]). The Cronbach’s alpha for this scale was 0.66, indicating acceptable levels of internal consistency for this scale in the current sample.

For children aged three years or older, mental health and behavioural difficulties were assessed using the Revised Rutter Parent Scale for Pre-school Children, specifically the 27-item ‘behavioural difficulties’ sub-scale ^26^. Of 201 children aged three or older, 197 (98%) had 12 or fewer missing items on this scale, and 175 of these 197 (89%) had no missing data. The total Rutter behavioural difficulties score for children with complete data was 17.6 (SD = 7.7; range = 3 - 41), of out a maximum possible 54. The prorated score for all 197 children with 12 or fewer missing items was 17.5 (SD = 7.8; range = 3 – 41), and was again calculated by taking the mean score of all items with data and then multiplying by the total number of items in the scale (27). Children with complete data had on average a marginally higher behavioural difficulties score than children with between one and 12 missing items (mean = 16.6; SD = 9.1; n = 22); a difference of 0.98 (95% CI: [-2.5; 4.5]). The Cronbach’s alpha for this scale was 0.86, indicating good levels of internal consistency for this scale in the current sample.

A summary of the child and parental worries questions are summarised in Table 5 (for children aged less than three years old) and Table 6 (for children aged three years or older); questions are split by child age as a different set of questions were asked to each age group. Some key findings include (all of which exclude ‘not applicable’ responses): 38 parents (19%) of children under three agreed/strongly agreed that they were worried about not having enough essential items for their child throughout the crisis; 104 (51%) agreed/strongly agreed that they were worried about the long-term impact of COVID-19 on their child’s future, and 77 (57%) agreed/strongly agreed that they were worried about their child returning to pre-school or nursery. For children aged three or older, 22 parents (12%) agreed/strongly agreed that their child was afraid to leave the house and 112 (61%) agreed/strongly agreed that they were worried about their child returning to school.

**Table 5.** Summary of child and parent worry questions, if child aged less than three years.

	Strongly disagree	Disagree	Neither agree nor disagree	Agree	Strongly agree	N/A
Child worried about catching COVID-19 or getting ill	35 (17%)	5 (2%)	10 (5%)	0 (0%)	0 (0%)	155 (76%)
Child worried about someone else catching COVID-19 or getting ill	36 (18%)	4 (2%)	9 (4%)	1 (0.5%)	0 (0%)	155 (76%)
Child seems afraid to leave the house right now	59 (29%)	9 (4%)	4 (2%)	2 (1%)	0 (0%)	131 (64%)
Child seems unsettled when doing usual activities (e.g., eating, sleeping, playing)	71 (35%)	26 (13%)	16 (8%)	6 (3%)	1 (0.5%)	86 (42%)
Parent worried child might transmit infection to someone else	44 (21%)	29 (14%)	39 (19%)	36 (17%)	8 (4%)	54 (26%)
Parent worried about not having enough food/milk/essential items for child during the outbreak	101 (48%)	40 (19%)	21 (10%)	28 (13%)	10 (5%)	9 (4%)
Parent worried about the long-term impact of COVID-19 on child’s future	35 (17%)	25 (12%)	40 (19%)	73 (35%)	31 (15%)	6 (3%)
Parent worried about the short-term impact of not taking child to social experiences/play groups/parks/nursery during crisis	19 (9%)	8 (4%)	14 (7%)	102 (49%)	61 (29%)	6 (3%)
Parent worried about child returning to nursery or pre- school if/when it opens	26 (12%)	13 (6%)	20 (10%)	37 (18%)	40 (19%)	74 (35%)

**Table 6.** Summary of child and parent worry questions, if child aged three years or older.

	Strongly disagree	Disagree	Neither agree nor disagree	Agree	Strongly agree	N/A
Child thinks that COVID-19 is a very serious issue	9 (5%)	6 (3%)	33 (17%)	77 (39%)	50 (25%)	23 (12%)
Child worried they will catch COVID-19	23 (12%)	37 (19%)	60 (30%)	42 (21%)	12 (6%)	24 (12%)
Child worried about someone else catching COVID-19 or getting ill	23 (12%)	12 (12%)	42 (21%)	63 (32%)	23 (12%)	24 (12%)
Child afraid to leave the house right now	75 (38%)	59 (30%)	26 (13%)	12 (6%)	10 (5%)	16 (8%)
Child worried they might transmit the infection to someone else	56 (28%)	43 (22%)	42 (21%)	22 (11%)	8 (4%)	27 (14%)
Child worried family won’t have enough food and other essential items during the outbreak	102 (52%)	34 (17%)	21 (11%)	12 (6%)	4 (2%)	25 (13%)
Child worried about missing school/work	54 (27%)	33 (17%)	30 (15%)	35 (185)	21 (11%)	25 (13%)
Child worried about the amount of money coming in	103 (52%)	28 (14%)	20 (10%)	7 (4%)	2 (1%)	38 (19%)
Child worried about the long-term impact this will have on their job prospects and the economy	95 (48%)	24 (12%)	17 (9%)	4 (2%)	3 (2%)	55 (28%)
Child is worried about not being able to see friends/attend social/sports activities	26 (13%)	13 (7%)	20 (10%)	67 (34%)	56 (28%)	17 (9%)
Parent worried about child returning to school if/when it open	23 (12%)	20 (10%)	29 (15%)	54 (27%)	58 (29%)	15 (8%)

### Schooling and social contacts

Details of the number of children attending school, pre-school or nursery, both in the whole sample and restricted to just those aged three or older, are presented in Table 7. At the time of questionnaire completion (26 ^th^ May to 5 ^th^ July) approximately one-in-six children were attending school/pre-school/nursery, increasing to one-quarter of children aged three or older.

**Table 7.** Descriptive statistics for school attendance and social contacts, split by age.

	Child currently going to school, pre- school or nursery		Child met any groups of 2 or more yesterday		Number of individual contacts met yesterday ^1^		
	Yes (%)	No (%)	Yes (%)	No (%)	N	Mean (SD)
Whole sample	63 (16%)	333 (84%)	57 (14%)	337 (86%)	289	3.3 (2)
Aged 3 or older	48 (25%)	143 (75%)	29 (15%)	160 (85%)	135	3.3 (2.2)
Aged less than 3	15 (7%)	190 (93%)	28 (14%)	177 (86%)	154	3.1 (1.9)

^1^ Note that the number of individual contacts excludes individuals met as part of a group. Additionally, in the whole G2 sample seven responses were of ‘11 or more’; here these have been coded as ‘11’.

The number of groups of two people or more that the child met the previous day is also described in Table 7. Approximately one-in-six children met a group on the previous day, with little difference between age groups. Of those who met a group, most only met one group (45; 79%), 8 (14%) met two groups, 1 (2%) met 3 groups, while 3 (5%) met 4 or more groups. The number of individual contacts (excluding those met in groups) is also displayed in Table 7. The number of individual contacts ranged between 1 and 11 (with 11 coded as ’11 or more’), with a mean of 3 contacts (and modal values of 2 and 3).

Details of where these group and individual contacts occurred are presented in Table 8. Most group meetings occurred in another home (32%), followed by the family home (22%), and then school (17%) and nursery (16%), with few group meetings taking place elsewhere. In contrast, the vast majority of individual contacts occurred at home (75%), with few individual contacts met in other locations.

**Table 8.** Descriptive statistics for location of child social contacts.

Location	Groups (of two or more; n = 63) ^1^	Individual contacts (n = 912) ^2^
Home	14 (22%)	682 (74.8%)
Work	0 (0%)	1 (0.1%)
School	11 (17%)	14 (1.5%)
Nursery	10 (16%)	11 (1.2%)
Park	5 (8%)	30 (3.3%)
Shops	1 (2%)	7 (0.8%)
Street	0 (0%)	21 (1.2%)
Another home	20 (32%)	124 (13.6%)
Another place	2 (3%)	22 (2.4%)

^1^ Includes details from up to three groups. ^2^ Note that the number of individual contacts excludes individuals met as part of a group. Includes detail from up to 10 individuals.

### Immunisations

Since the beginning of lockdown 57 children (14% of the 394 who answered the question) were due routine vaccinations. Of these 57, 49 (86%) received these vaccinations, while 8 (14%) did not, with reasons for missing the vaccination including: being worried about COVID-19 and deciding to wait, not receiving an invite from their GP, and various ‘other’ reasons (including not being able to book an appointment, child not allowed live vaccines for health reasons, and personal beliefs/decisions not to vaccinate).

### Assessment of potential selection bias

**Figure 2.**
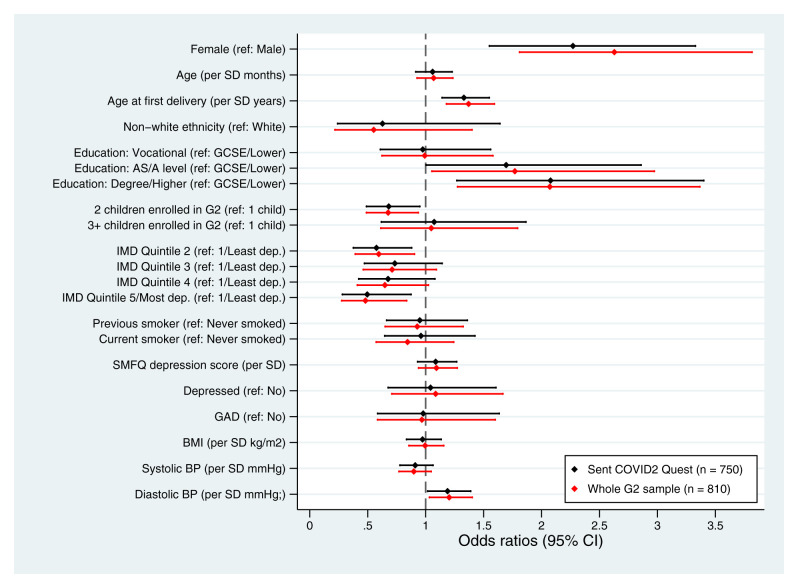
Forest plot describing the factors predicting G2 questionnaire completion. This plot assesses questionnaire completion in both G1 parents sent a questionnaire (n=750; n completed = 289; n not completed = 461) and all known G1 parents enrolled in ALSPAC-G2 (n=810; n completed = 289; n not completed = 521). All results are odds ratios from logistic regression models with ‘completing the G2 questionnaire’ as the outcome. All models are unadjusted univariable models. Continuous variables have been standardised over one standard deviation to facilitate comparisons between different continuous variables. Results to the right of the dashed line indicate increased odds of completing the G2 questionnaire, either relative to the reference category for categorical variables, or per one unit standard deviation increase for continuous variables. Note that all variables refer to G1 parental characteristics (sex, age, etc.). Sources of data: Ethnicity (G0 pregnancy questionnaire); Highest education qualification (recent G1 questionnaire or ALSPAC-G2 enrolment questionnaire); Index of multiple deprivation (from linkage data based on address data held by ALSPAC at most recently-available date [January 2014]); Smoking status (from most recent questionnaire or clinic that individual completed/attended, back to age 17/18); Short Moods and Feeling Questionnaire (SMFQ) ^37^ continuous depression score (from most recent questionnaire completed, back to age 21/22); Depression and generalised anxiety disorder (from revised Clinical Interview Schedule [CIS-R] questionnaire ^38^, either at most recent clinic [age 24], or from age 17/18 clinic if missing at age 24); BMI and blood pressure (from most recent clinic that the participant attended). Sample sizes (sent a questionnaire; all known G1 parents): sex ( *n*=750; *n*=810); age ( *n*=750; *n*=810); age at first delivery ( *n*=744; *n*=803); ethnicity ( *n*=630; *n*=676); education ( *n*=544; *n*=559); number of children enrolled in ALSPAC-G2 ( *n*=744; *n*=803); IMD ( *n*=695; *n*=749); smoking status ( *n*=710; *n*=752); SMFQ continuous depression score ( *n*=657; *n*=686); CIS-R depression diagnosis ( *n*=599; *n*=630); GAD CIS-R diagnosis ( *n*=598; *n*=629); BMI ( *n*=689; *n*=724); blood pressure ( *n*=669; *n*=701).

We conducted two analyses to explore whether any sociodemographic factors were associated with G2 questionnaire completion among G1 parents; the first compared G2 questionnaire completion among those who were sent the questionnaire (n=750; comparison of 289 who completed vs 461 who did not), while the second compared G2 questionnaire completion among all known G1 parents enrolled in ALSPAC-G2, including the 60 G1 parents who were not sent a COVID2 questionnaire (n=810; 289 who completed vs 521 who did not). In both analyses the 42 respondents who began the G0/G1 questionnaire but did not complete the G2 questionnaire are in the ‘not completed’ group. Results of these unadjusted models are displayed in Figure 2.

Results from both analyses were largely consistent with each other. Completion of the questionnaire was more likely in female than male parents, in those who were older when their first child was born, and was structured by socioeconomic position, with increased deprivation and lower educational attainment associated with lower rates of completion. Parents with two children enrolled in ALSPAC were slightly less likely to have data relative to parents with only one child enrolled, although no difference was found for parents with three or more enrolled children. Smoking status, recent mental health issues, and physical health (as inferred from BMI and blood pressure) had little association with questionnaire completion (other than for diastolic blood pressure, where higher values were associated with increased odds of completion).

## Strengths and limitations of the data

There are a number of strengths of this data collection. This is one of the first prospective cohort studies to collect data on children’s response to the COVID-19 pandemic. The three-generational nature of ALSPAC and the depth of phenotypic data available is unique and unparalleled; the availability of repeat data to link pandemic data with pre-pandemic baseline measures allows assessment of longitudinal change in children’s health and wellbeing. For example, we have already been able to demonstrate the impact the pandemic has had on adult mental health in the G0 and G1 generations ^24^, and research is currently underway to explore changes among the G2 child generation. Future G2 data collections will be able to explore the long-term developmental consequences of the pandemic, the factors predicting resilience to this crisis, as well as linking to ongoing observed recordings of family interactions at home.

These data will also be used to describe children’s contact patterns to help inform estimates of the COVID-19 Reproduction number and improve the predictive accuracy of epidemic models. Currently, data on children’s contact patterns are very limited ^27, 31, 32^, despite their use in mathematical models to design and determine effective infectious disease control strategies (for example, school-based vaccination programmes).

A key limitation of this data collection is the relatively small sample size of 411 G2 children with data (linked to only 289 G1 parents). This small sample size limits the power of many analyses, meaning that this data may be underpowered to detect subtle, but potentially important, effects. This issue is exacerbated if analyses are stratified by age; for instance, there are only 201 children aged three or over.

Another limitation is that the response rate was non-random with regard to sex and socio-economic status (see Figure 2), resulting in both an unrepresentative sample and potential issues of selection/collider bias ^33, 34^. Although completion was non-random, the proportion of known G1 parents enrolled in ALSPAC-G2 who were sent a questionnaire was high (750 out of 810; 93%), and individuals who were not sent a questionnaire appeared broadly similar to participants who were sent a questionnaire but did not complete it. This suggests that any additional issues of selection bias based on being sent a questionnaire may be minimal, although male G1 parents did appear more likely to not be sent a questionnaire. In addition, in families with more than one G2 child, older children are more likely to have completed questionnaire data ( Table 2), potentially leading to further bias as data is more likely to be missing for younger siblings. Several additional sources of potential selection bias are possible, beyond those identified above. For instance, not all known G1 parents are enrolled in ALSPAC-G2 ^20^, inclusion in ALSPAC-G2 is restricted to ALSPAC-G1 participants (other than for their partners), and many G1 participants who have since been lost to follow-up are also likely to be parents, but this information is unknown to ALSPAC. This bias is further amplified as ALSPAC-G2 is an open cohort and the G1 parents are still young, meaning that the parental age will be younger than average, and hence also not representative of the wider population. For additional discussion on issues of selection bias in this cohort, see the ALSPAC-G2 cohort profile ^20^. While we have provided a brief assessment of potential selection bias regarding G2 COVID questionnaire completion here, we stress that the impact of selection bias and analyses to explore it will depend on the specific research question being addressed, and do not dictate how researchers using this resource should analyse this data. To boost response rates and alleviate potential bias, ALSPAC are actively developing and implementing methods to encourage participation of these ‘disengaged’ participants.

ALSPAC recruited participants from one geographical area, of mostly White European families, and several selection processes (see above) have influenced who is included in the G2 COVID-19 dataset, meaning that results from this dataset may not generalise to the wider UK population or non-UK populations. However, as ALSPAC is part of several collaborative efforts we would recommend, where appropriate, using these data alongside other relevant cohort data with similar measurements, for example the Born in Bradford data ^35^. Additionally, while we make no claims about representativeness, by using longitudinal data it is possible to assess changes over time within individuals in this cohort, allowing both pre- vs post-COVID comparisons, as well as exploring short- vs long-term responses to the pandemic (e.g., see 24, 36).

While to some extent unavoidable, a further limitation is the potential for measurement bias; as these questionnaires were completed by parents, answers may reflect the parents’ perceived behaviour of their child in response to the COVID-19 pandemic, rather than the child’s actual behaviour (e.g., a parent who is very concerned about the potential impact of the pandemic on their child might perceive a larger change in their child’s behaviour). We also acknowledge that the COVID-19 case status data likely contains a high degree of measurement error as many children with COVID-19 will have been asymptomatic, few will have been tested, and the symptom checklist is likely to lack sensitivity and specificity in children ^7, 30^. We aim to provide more accurate measures of COVID-19 status in the future using a combination of serological testing and data linkage (while noting that these sources of data may contain some degree of measurement error and potential false positives due to a lack of test sensitivity and possible antibody level decline over time).

In summary, data from this questionnaire aimed to assess how children have responded to the COVID-imposed lockdown, the impact of the lockdown on their behaviour, health and emotional well-being, and their contact patterns during the pandemic. These ALSPAC data have the potential to contribute to policy-relevant evidence for the future management of the pandemic and the health and well-being of children who have been exposed to it and its management. These data are available for researchers as described below.

## Consent

Completion of the questionnaire was optional and choosing to complete the questionnaire is considered informed consent for the questionnaire.

Ethical approval for the study was obtained from the ALSPAC Ethics and Law Committee and the Local Research Ethics Committees. Informed consent for the use of data collected via questionnaires and clinics was obtained from participants following the recommendations of the ALSPAC Ethics and Law Committee at the time. Study participants have the right to withdraw their consent for elements of the study or from the study entirely at any time. Full details of the ALSPAC consent procedures are available on the study website.

## Data availability

### Underlying data

ALSPAC data access is through a system of managed open access. The steps below highlight how to apply for access to the data included in this data note and all other ALSPAC data:

1. Please read the ALSPAC access policy, which describes the process of accessing the data and samples in detail, and outlines the costs associated with doing so.

2. You may also find it useful to browse our fully searchable research proposals database, which lists all research projects that have been approved since April 2011.

3. Please submit your research proposal for consideration by the ALSPAC Executive Committee. You will receive a response within 10 working days to advise you whether your proposal has been approved.

Please note that a standard COVID-19 dataset will be made available at no charge (see description below); however, costs for required paperwork and any bespoke datasets required additional variables will apply.

### Extended data

Open Science Framework: ALSPAC COVID-19 First and Second Questionnaire, https://doi.org/10.17605/OSF.IO/74GBJ ^39^.

This project contains the following extended data:

1. The final questionnaire
2. List of variable names and labels
3. Associated data dictionary including frequencies of all variables that are available

Data are available under the terms of the Creative Commons Attribution 4.0 International license (CC-BY 4.0).

### ALSPAC-G2 COVID-19 Questionnaire Data File

Data from the ALSPAC-G2 COVID-19 questionnaire is available in two ways.

1. A freely available standard set of data together with key sociodemographic variables (where available) is available on request (see data availability section). Subject to the relevant paperwork being completed (costs may apply to cover administration) this dataset will be made freely available to any bona fide researcher requesting it. Note that this data has a random ID and cannot be linked to existing ALSPAC data. Variable names will follow the format *covid2_g2_xxxx* where *xxxx* is a four-digit number. A full list of variables released is available here: https://osf.io/xwqgv/. Frequencies of variable and details of any coding/editing decisions and derived variables are also available in the data dictionary: https://osf.io/usbnd/.

2. A formal release file has been created for ALSPAC-G2 participants in the usual way and now forms part of the ALSPAC resource, and can be linked to existing G2, G1 and/or G0 data. This dataset (or sections therein) can be requested in the usual way. Variable names will replicate those in 1) above.

Text data and other potentially disclosive information will not be released until they have been coded appropriately. Table 9 describes the data that is withheld at the time of first release. Data will be incorporated back into both file sets as they become available.

**Table 9.** Data from questions that will not be released until coded.

Question number	Question text
**Section 2: School**	
2a1	What else child likes about not going to school
2a2	What else child misses about not going to school
**Section 3: Contacts**	
3c1/2/3	Description of group 1/2/3 that child met with yesterday
3c_other	Details of other groups that child met with yesterday
3d1/2/3/4/5/ 6/7/8/9/10	Description of individual person 1/2/3/4/5/6/7/8/9/10 that child met yesterday
3d_other	Details of other individuals that child met with yesterday
**Section 4: Your Child’s Health**	
4b22	Where on the body were the unexplained rashes?
4b25	Details of other medical attention (if sought in past week)
4d2	Other reason(s) for not receiving scheduled vaccinations
