# Supplementary material for: The Avon Longitudinal Study of Parents and Children - A resource for COVID-19 research: Generation 2 questionnaire data capture May-July 2020
Source: Wellcome Open Res. 2021 Apr 14;5:278. Originally published 2020 Nov 26. [Version 2] doi: 10.12688/wellcomeopenres.16414.2 (PMC7968471; doi:10.12688/wellcomeopenres.16414.2)
Supplement: Peer review history

# Peer review: Reviewer response for version 2

**DOI:** 10.21956/wellcomeopenres.18506.r43490

**Published:** 2021-04-15

**Authors:** Deepa Dongarwar

**Affiliations:** 1 Center of Excellence in Health Equity, Training, and Research, Baylor College of Medicine, Houston, Texas, USA

The authors have sufficiently addressed all the comments. The data note can be approved in its current form.

Are sufficient details of methods and materials provided to allow replication by others?

No

Is the rationale for creating the dataset(s) clearly described?

Yes

Are the datasets clearly presented in a useable and accessible format?

No

Are the protocols appropriate and is the work technically sound?

Yes

Reviewer Expertise:

Epidemiology, data science, Maternal and Child Health, machine learning.

I confirm that I have read this submission and believe that I have an appropriate level of expertise to confirm that it is of an acceptable scientific standard.

# Peer review: Reviewer response for version 1

**DOI:** 10.21956/wellcomeopenres.18060.r42756

**Published:** 2021-03-15

**Authors:** Emily E Cameron

**Affiliations:** 1 Department of Psychology, University of Manitoba, Manitoba, Canada

Thank you for the opportunity to review the manuscript titled “The Avon Longitudinal Study of Parents and Children – A resource for COVID-19 research: Generation 2 questionnaire data capture May-July 2020.” The manuscript provides a description of a modified questionnaire for the existing ALSPAC birth cohort to collect data on the impact of COVID-19 on parents and children. Given the methodology, this study design allows for valuable comparisons pre- and post-pandemic, as well as longitudinal follow-up with families. This manuscript is well-written, clear, and concise. The authors have provided a clear rationale and need for the study. The resulting information will be helpful for continued tracking of the impact of COVID-19 on child development, which is an understudied area with longitudinal and existing cohorts at present. The manuscript is scientifically sound at present. I have a few minor comments to consider for clarity.

1. Methods: The study authors state that “Of these 1,116 G2 children, 455 (41%) are from families with only 1 child enrolled in ALSPAC-G2, 476 (43%) have one other sibling enrolled, 153 (14%) have two other siblings enrolled, and 32 (3%) have three or more other siblings enrolled.” Is there data on how many siblings in families were not enrolled in the study or does this refer to all children of parents enrolled in the study?
2. Content: It would be helpful to note clearly in the bulleted list which measures/questions were created by the authors (vs. standardized measures).
3. Response rate: Is the response rate for the COVID-19 survey consistent with previous ALSPAC surveys?
4. It would be helpful to note any future data collection plans that may be similar or improved based on the current findings and considerations. In particular, are there opportunities to improve the response rate with the families who did not previously participate?

Are sufficient details of methods and materials provided to allow replication by others?

Yes

Is the rationale for creating the dataset(s) clearly described?

Yes

Are the datasets clearly presented in a useable and accessible format?

Yes

Are the protocols appropriate and is the work technically sound?

Yes

Reviewer Expertise:

Clinical psychology; child development; parental mental health; parenting; family functioning.

I confirm that I have read this submission and believe that I have an appropriate level of expertise to confirm that it is of an acceptable scientific standard.

# Response

**Published:** 2021-03-30

**Authors:** Daniel Smith

**Affiliations:** University of Bristol, UK

We thank the reviewer for their positive comments and for the helpful suggestions which have strengthened the manuscript and improved clarity in key areas. The reviewer’s comments are below, with our responses in italics underneath.

 Methods: The study authors state that “Of these 1,116 G2 children, 455 (41%) are from families with only 1 child enrolled in ALSPAC-G2, 476 (43%) have one other sibling enrolled, 153 (14%) have two other siblings enrolled, and 32 (3%) have three or more other siblings enrolled.” Is there data on how many siblings in families were not enrolled in the study or does this refer to all children of parents enrolled in the study?

*This is an excellent question. ALSPAC strives to enrol all children – and hence all siblings – into the G2 study, and to the best of our knowledge there are less than a handful of instances where, if siblings are known, one or more sibling is enrolled but another is not. An important caveat here is if we lose contact with parents and they do not inform us of later-born children; these children would remain unknown to ALSPAC (although we would enrol them if/when we are made aware of them). However, we can say that at the time children were enrolled in the G2 study, in almost all cases known siblings were enrolled as well.*

**

*As this is an important point, we have now added additional explanation of this in the Methods section: “In nearly every instance where siblings are known, all siblings have been enrolled into ALSPAC-G2 (with fewer than a handful of known exceptions). However, it is important to note that later-born siblings would remain unknown to ALSPAC if contact is lost with parents after enrolment.”*

 Content: It would be helpful to note clearly in the bulleted list which measures/questions were created by the authors (vs. standardized measures).

*Another excellent idea. Details of provenance for each measure have now been added to the list of topics.*

**

*This updated information includes:*

- *Section 1: Whether child has a regular routine (these questions were adapted from the Co-SPACE study)*
- *Section 1: Child temper tantrums and parent’s response to bad behaviour (non-standardised measures previously developed by ALSPAC)*
- *Section 2: Whether child is currently attending school, pre-school or nursery (non-standardised measure)*
- *Section 2: If the child is not going to school, what they like about not going to school and what they miss about not going to school (non-standardised measure)*
- *Section 3: Social contacts and methods of communication (these questions were adapted from the Co-SPACE study)*
- *Section 3: Time spent with various family members and friends (non-standardised measure previously developed by ALSPAC)*
- *Section 4: Whether child has/has had COVID-19 (non-standardised measure)*
- *Section 4: Missed vaccinations as a result of COVID-19 (non-standardised measure)*

 

 Response rate: Is the response rate for the COVID-19 survey consistent with previous ALSPAC surveys?

*This is another important point, but providing a definitive answer is unfortunately quite difficult for a number of reasons. First, the G2 questionnaire was embedded within a wider G0/G1 questionnaire, making comparisons difficult (as typically G2 questionnaires are stand-alone data collections). Secondly, in response to COVID-19 the ALSPAC questionnaires were only sent to individuals with a known email address (who may be more engaged with the study, as they keep their details up-to-date), and there was no monetary incentive to complete the questionnaire (unlike most other ALSPAC questionnaires which are also completed in paper). Thirdly, this G2 questionnaire was asked as part of the main G0/G1 questionnaire, so potentially questionnaire fatigue may have set in by this point. Finally, the overall impact of the COVID-19 pandemic on response rates is difficult to know (response may be higher as the results are ‘for a wider cause’; or response may lower, due to COVID-related time constraints, stress or other priorities).*

**

*Nonetheless, with these caveats in mind, we can say the following. First, the response rate to the wider G0/G1 questionnaire was identical for both G1 parents enrolled in the ALSPAC-G2 study and for G1 participants not enrolled in ALSPAC-G2 as a parent (44% in both). Second, the G1 response rate to this second COVID-19 questionnaire was lower than the response rate to the first COVID-19 questionnaire (44% vs 51%). Third, this response rate of 44% is generally consistent with other recent G1 questionnaires (return rate of 42-48% of those sent a questionnaire). This suggests that response rates are broadly consistent with previous G1 ALSPAC surveys, albeit slightly lower than the first COVID-19 questionnaire.*

**

*We have added this information to the ‘response rate’ section: “Of these 750 parents, 331 (44%) returned the main G0/G1 questionnaire, a response rate identical to that of G1s not enrolled in ALSPAC-G2 (44%; 2,380/5,398). While this response rate is lower than the G1 response rate to the first ALSPAC COVID-19 questionnaire (51%) ^22^, it is typical of other recent G1 questionnaires with response rates ranging between 42% and 48%.”*

 It would be helpful to note any future data collection plans that may be similar or improved based on the current findings and considerations. In particular, are there opportunities to improve the response rate with the families who did not previously participate?

*Another excellent point. This is again hard to answer definitively as there are lots of unknowns due to the COVID-19 pandemic; it is difficult to know what factors are important in determining response rates and how best to promote participation. For instance: the ‘online only’ format may be an issue as we may miss those without an email account (or those who do not check their email regularly); some individuals may not respond due to time-constraints if they are a healthcare/key worker (the reverse may also be true; individuals working fewer hours, or on furlough, may have more free time than usual to complete the questionnaire); additional stresses (e.g., financial, health, childcare, family) due to COVID-19 may lower participation rates; the lack of monetary incentive may also have put some participants off completing. Understanding the factors associated with response – especially COVID-19-related factors – is therefore difficult; recommending strategies to enhance participation even more so.*

**

*Nonetheless, the results of the selection pressures analysis (figure 2) suggest that we need to target male participants, those who were younger at first delivery, those with lower educational attainment, and individuals from more deprived areas. These socioeconomic position-related pressures are common to the wider ALSPAC cohort, as well as to other cohort studies, and can be difficult to shift. ALSPAC is currently implementing strategies to improve participation among certain groups (e.g., to improve the response rates of male participants), and is working hard on developing a strategy to encourage ‘disengaged’ participants to participate in future ALSPAC data collections, in particular the up-coming ‘@30’ clinic.*

**

*We have included some additional details of this in the discussion: “To boost response rates and alleviate potential bias, ALSPAC are actively developing and implementing methods to encourage participation of these ‘disengaged’ participants.”*

# Peer review: Reviewer response for version 1

**DOI:** 10.21956/wellcomeopenres.18060.r42757

**Published:** 2021-03-01

**Authors:** Deepa Dongarwar

**Affiliations:** 1 Center of Excellence in Health Equity, Training, and Research, Baylor College of Medicine, Houston, Texas, USA

This is a well conducted study with rich data obtained from 3 generations. The manuscript is well-written with minimal errors. There are few concerns that need to be addressed.

  As this manuscript will be read in future when the pandemic is over, authors should provide a reference date in their first line of the introduction. E.g. “As of February 27, 2021, the coronavirus disease…”.

 Instead of saying “with only a minority of cases progressing to disease”, it should be something like “with only a minority of cases progressing to severe form of the disease”.

 Citation #16 in introduction should be in superscript. Please write in a form “except a study which was longitudinal in nature ^16^”

 Table footnotes state that information presented in the table is based on available information. It is unclear what the sample size was for some of the continuous variable, e.g. BMI, which makes the replication of the methods difficult. More details should be included.

 The superscripts 1 and 2 in table 4 seem to have been interchanged. Correct them.

 Instead of presenting the information in table 5 and 6, which contains a lot of information, the authors could create a scoring system by maybe combining the disagree categories, the agree categories etc. With all these numbers, it is very difficult to read and understand the main points of the table. Furthermore, combining the categories will reduce the small sample size in some of these categories.

Are sufficient details of methods and materials provided to allow replication by others?

No

Is the rationale for creating the dataset(s) clearly described?

Yes

Are the datasets clearly presented in a useable and accessible format?

No

Are the protocols appropriate and is the work technically sound?

Yes

Reviewer Expertise:

Epidemiology, data science, Maternal and Child Health, machine learning.

I confirm that I have read this submission and believe that I have an appropriate level of expertise to confirm that it is of an acceptable scientific standard, however I have significant reservations, as outlined above.

# Response

**Published:** 2021-03-30

**Authors:** Daniel Smith

**Affiliations:** University of Bristol, UK

We thank the reviewer for their supportive and constructive comments, and we hope that our revisions have addressed all of the concerns raised. The reviewer’s comments are below, with our responses in italics below.

 As this manuscript will be read in future when the pandemic is over, authors should provide a reference date in their first line of the introduction. E.g. “As of February 27, 2021, the coronavirus disease…”.

*This is a very good point, thanks for suggesting it. We have now amended the first sentence to read “As of 30 ^th^ March 2021, the coronavirus disease 2019 (COVID-19) pandemic continuous to be a rapidly developing global health challenge.”*

 Instead of saying “with only a minority of cases progressing to disease”, it should be something like “with only a minority of cases progressing to severe form of the disease”.

*This text has now been amended as suggested.*

 Citation #16 in introduction should be in superscript. Please write in a form “except a study which was longitudinal in nature ^16^”

*This is a formatting decision of the journal which we have no control over, so cannot change this.*

 Table footnotes state that information presented in the table is based on available information. It is unclear what the sample size was for some of the continuous variable, e.g. BMI, which makes the replication of the methods difficult. More details should be included.

*Details of sample size for each variable have now been added to both table 3 and the figure 2 legend.*

 The superscripts 1 and 2 in table 4 seem to have been interchanged. Correct them.

*Good spot, thanks for noticing. This has now been updated.*

 Instead of presenting the information in table 5 and 6, which contains a lot of information, the authors could create a scoring system by maybe combining the disagree categories, the agree categories etc. With all these numbers, it is very difficult to read and understand the main points of the table. Furthermore, combining the categories will reduce the small sample size in some of these categories.

*We thank the reviewer for their suggestion. However, after discussion between the authors we feel that these tables should be left as they are. Combining categories results in a loss of information; as this is a Data Note we believe that we should present all of the available information, so that users of this resource can understand the raw data. Users of this data can of course collapse these categories to simplify comparisons and boost the sample size of certain categories, but we do not believe that this would be appropriate for a Data Note.*

# Peer review: Reviewer response for version 1

**DOI:** 10.21956/wellcomeopenres.18060.r41918

**Published:** 2021-02-10

**Authors:** Duncan Astle

**Affiliations:** 1 MRC Cognition and Brain Sciences Unit, University of Cambridge, Cambridge, UK

I have little to say, because I think this is a well-constructed and highly useful overview of everything included in the latest ALSPAC COVID-19 questionnaire data. It is incredibly rare to have the opportunity to access data from individuals across three generations, with such rich data in order to contextualise the impact of recent changes like the COVID pandemic. For this reason I would regard this as a unique resource. As I read, there were of course limitations inherent in the data available, but these are all mentioned by the authors themselves at the end of this article. I had only two main recommendations. The first is that I would have started the results section with information about biases in who was recruited in this latest wave. As I read the results I kept wondering which slice of the original cohort we were looking at, because of the biases that may have crept in with the re-recruitment. In the end it turns out that the subset who completed the questionnaire are fairly representative of the original cohort, so I would let reader know this up front. Secondly, I wondered whether there were any better measures of mental health (especially for the benchmarking of any biases) – the categorical presence of GAD or Depression, for example may be relatively coarse. I wondered whether there were any more sensitive measures of mental health that could be used in the demonstrate how representative this subset of the cohort are. But other than that, I think this was a really great piece of work, and applaud the authors for putting this together. I am sure it will be incredibly useful to those considering accessing the latest haul of useful ALSPAC data.

Are sufficient details of methods and materials provided to allow replication by others?

Yes

Is the rationale for creating the dataset(s) clearly described?

Yes

Are the datasets clearly presented in a useable and accessible format?

Yes

Are the protocols appropriate and is the work technically sound?

Yes

Reviewer Expertise:

Studies of child development, using a wide range of different data types, including large scale studies like ALSPAC.

I confirm that I have read this submission and believe that I have an appropriate level of expertise to confirm that it is of an acceptable scientific standard.

# Response

**Published:** 2021-03-30

**Authors:** Daniel Smith

**Affiliations:** University of Bristol, UK

We are delighted with the incredibly positive review and thank the reviewer for their constructive comments. We reply to the reviewer’s suggestions in turn below.

*Starting the results section with information about biases:* We completely agree that understanding potential biases in the data is incredibly important and a key piece of information to help interpret the results. However, as this is a Data Note (rather than a standard research paper) we – and the publishers – believe that the focus should be on the data itself, with the assessment of potential biases presented afterwards. We have now added a sentence to the beginning of the results section (‘Key results – sociodemographic characteristics’) informing readers that an assessment of bias will be given later in the results section: “For an assessment of potential biases in parents who completed the questionnaire, compared to the wider G2 parent cohort, see the section ‘Assessment of potential selection bias’ below.”

*More sensitive measures of mental health: *We have now added an additional continuous measure of depression to the assessment of potential selection bias plot (figure 2). This is based on the Short Moods and Feelings Questionnaire (SMFQ) total score. This scale has been asked repeatedly in the G1 cohort, with the most recent SMFQ total score (going back to age 21) used here. The results of this finer-grained measure are comparable to the coarser binary depression variable based on the CIS-R questionnaire, with no association between total SMFQ score and questionnaire completion. Note that we are only able to assess depression on a continuous scale as the SMFQ has been asked repeatedly over recent years; the most recent continuous anxiety scale (the GAD-7 questionnaire) was collected several years ago when the G1 participants were aged 21/22, and also contains missing data on approximately half of the G2 parent cohort, so would not be appropriate to include here.
